# An application of partial least squares for identifying dietary patterns in bone health

**DOI:** 10.1007/s11657-017-0355-y

**Published:** 2017-07-12

**Authors:** Tiffany C. Yang, Lorna S. Aucott, Garry G. Duthie, Helen M. Macdonald

**Affiliations:** 1 Department of Health Sciences, University of York, Seebohm Rowntree, York, YO10 5DD UK; 2 Health Sciences Building, University of Aberdeen, Foresterhill, Aberdeen, AB25 2ZD UK; 3 Natural Products Group, Rowett Institute of Nutrition and Health, University of Aberdeen, Aberdeen, AB21 9SB UK

**Keywords:** Bone mineral density, Dietary patterns, Partial least-squares, Postmenopausal women

## Abstract

**Summary:**

In a large cohort of older women, a mechanism-driven statistical technique for assessing dietary patterns that considers a potential nutrient pathway found two dietary patterns associated with lumbar spine and femoral neck bone mineral density. A “healthy” dietary pattern was observed to be beneficial for bone mineral density.

**Introduction:**

Dietary patterns represent a broader, more realistic representation of how foods are consumed, compared to individual food or nutrient analyses. Partial least-squares (PLS) is a data-reduction technique for identifying dietary patterns that maximizes correlation between foods and nutrients hypothesized to be on the path to disease, is more hypothesis-driven than previous methods, and has not been applied to the study of dietary patterns in relation to bone health.

**Methods:**

Women from the Aberdeen Prospective Osteoporosis Screening Study (2007–2011, *n* = 2129, age = 66 years (2.2)) provided dietary intake using a food frequency questionnaire; 37 food groups were created. We applied PLS to the 37 food groups and 9 chosen response variables (calcium, potassium, vitamin C, vitamin D, protein, alcohol, magnesium, phosphorus, zinc) to identify dietary patterns associated with bone mineral density (BMD) cross-sectionally. Multivariable regression was used to assess the relationship between the retained dietary patterns and BMD at the lumbar spine and femoral neck, adjusting for age, body mass index, physical activity level, smoking, and national deprivation category.

**Results:**

Five dietary patterns were identified, explaining 25% of the variation in food groups and 77% in the response variables. Two dietary patterns were positively associated with lumbar spine (per unit increase in factor 2: 0.012 g/cm^2^ [95% CI: 0.006, 0.01]; factor 4: 0.007 g/cm^2^ [95% CI: 0.00001, 0.01]) and femoral neck (factor 2: 0.006 g/cm^2^ [95% CI: 0.002, 0.01]; factor 4: 0.008 g/cm^2^ [95% CI: 0.003, 0.01)]) BMD. Dietary pattern 2 was characterized by high intakes of milk, vegetables, fruit and vegetable juices, and wine, and low intakes of processed meats, cheese, biscuits, cakes, puddings, confectionary, sweetened fizzy drinks and spirits while dietary pattern 4 was characterized by high intakes of fruits, red and white meats, and wine, and low intakes of vegetables and sweet spreads.

**Conclusion:**

Our findings using a robust statistical technique provided important support to initiatives focusing on what constitutes a healthy diet and its implications.

**Electronic supplementary material:**

The online version of this article (doi:10.1007/s11657-017-0355-y) contains supplementary material, which is available to authorized users.

## Introduction

Osteoporosis is a global health issue that affects millions of individuals around the world, with fragility and fracture resulting from reduced bone mass or increased micro-architectural deterioration of the bone tissue [1]. Nutritional intake has long been considered to have an important role in bone mineral density (BMD) and maintenance through various mechanisms, including alterations in the endocrine system or in bone metabolism and structure [2, 3]. Traditionally, individual foods or nutrients were singled out, and most research has focused on the roles of certain vitamins or minerals. This approach with single nutrients or foods, while valuable, is problematic due to its inherent reductionist nature and lack of dietary context [4, 5].

Dietary patterns provide a more complete perspective to health and disease, as these patterns would more closely mirror what occurs in the real world where individuals eat meals of combined foods and nutrients. The combination of multiple nutrients utilized in a dietary patterns analysis may therefore be more powerful in detecting effects, as the influence of a single nutrient may be too small and the resulting recommendations too difficult to follow [6]. Examining dietary patterns could have a greater public health importance, as evaluating the overall combinations of food intake would be more practical and easily translatable to the public and has been advocated by the US Dietary Guidelines Advisory Committee [7].

The relationship between dietary patterns and bone health has been previously explored through data reduction methods such as dietary indices like the Mediterranean diet [8], factor and principal component analysis (PCA) [9, 10], reduced rank regression (RRR) [11] and cluster analysis [12]. However, though data-reduction methods such as PCA and RRR that are more commonly used, they may not be ideal in selecting dietary patterns in relation to disease because of how they reduce dietary data. PCA derives a set of uncorrelated factors (dietary patterns) characterized by the different foods through explanation of variation in the food intake with no consideration for the outcome measure [4]. RRR is similar to PCA in deriving uncorrelated factors, but has a different goal: dietary patterns are derived to account for the variation not in food intake, but in a set of response variables which are nutrients or biomarkers thought to be important to the health or disease outcome [13].

Partial least squares (PLS) is a mix between PCA and RRR, where extracted dietary patterns account for variation in both dietary intake and the intermediary response variables related to the health or disease outcome [13]. Dietary patterns derived by maximizing the variance in both food groups (the “predictors”) and the outcome-related nutrients or biomarkers (the “response variables”) may be more suitable as it would allow for directed data reduction of food groups through specific nutrients or biomarkers of interest. Common in bioinformatics and chemometrics research, PLS has not been widely utilized in epidemiology, and not in relation with bone health. It is hypothesized to be a more useful method of deriving dietary patterns because it considers the potential biochemical pathways by which the dietary patterns may influence health [14].

Our objective was to identify dietary patterns through the novel use of partial least-squares analysis using nutrients hypothesized to influence bone health as the intermediary response variables. We then assessed and evaluated the relationship between these derived dietary patterns and BMD in a population of women in northeast Scotland.

## Methods

### Subjects

Subjects are women from the Aberdeen Prospective Osteoporosis Screening Study (APOSS) cohort, a population-based screening program for assessing osteoporotic fracture risk, which initially recruited 5119 women aged 45–54 years between 1990 and 1994 using random selection from Community Health Index [15]. No exclusion criteria were applied, as baseline participants were recruited for a population-based screening program for osteoporosis fracture risk. We utilized information from third (2007–2011; *n* = 2129) follow-up visit. At this visit, subjects were given questionnaires to evaluate risk factor assessment and dietary intake through food frequency questionnaires (FFQ) and completed bone densitometry scans.

Participants were weighed in kilograms (kg) on calibrated balance scales (Seca, Hamburg, Germany) while wearing light clothing and no shoes. Heights were measured in centimetres (cm) using a stadiometer (Holtain Ltd., Crymych, UK). Body mass index (BMI) was calculated as weight in kilograms divided by height in metres squared.

Usual dietary intake over the previous 12 months was assessed using a 98-question semi-quantitative FFQ based on the Caerphilly FFQ and modified to include more detail on foods commonly consumed in northeast Scotland. It has been validated using 7-day weighed food records and biochemical markers of antioxidant status, including ascorbic acid, carotene, retinol, and α- and γ-tocopherol [16, 17]. Intakes of specific nutrients were calculated using the UK’s McCance and Widdowson’s composition of foods [18] based on the weight of each food consumed multiplied by the frequency of intake with its assigned portion size. All food and beverage items were grouped into 37 food groups on the basis of similarities of food and nutrient composition (Appendix Table 1).

**Table 1:** Characteristics of the Aberdeen Prospective Osteoporosis Screening Study

	Number	Mean (SD)/%
Age (years)	2129	66.0 (2.2)
BMI (kg/m^2^)	2122	27.9 (4.9)
Physical activity level^a^	1681	1.7 (0.2)
Current smokers	188	8.9
Menopausal status		
Pre-menopausal	–	–
Peri-menopausal	–	–
Post-menopausal, no HRT	871	41.0
Post-menopausal, past HRT	1259	59.0
Post-menopausal, current HRT	–	–
National Deprivation Category^b^		
1	578	27.3
2	895	42.2
3	170	8
4	288	13.6
5	143	6.8
6	45	2.1
Bone mineral density (g/cm^2^)		
Lumbar spine	2097	1.09 (0.17)
Femoral neck	2021	0.93 (0.12)
Dietary intakes	Number	Median (IQR)
Energy intake (MJ/day)	1681	10.1 (9.5, 10.9)
Alcohol (g/day)	1675	4 (0, 9)
Protein (g/day)	1675	78 (65, 91)
Vitamin C (mg/day)	1675	138 (95, 186)
Vitamin D (μg/day)	1675	3 (2, 4)
Calcium (mg/day)	1675	996 (820, 1191)
Phosphorus (mg/day)	1675	1403 (1176, 1652)
Potassium (mg/day)	1675	3389 (2880, 3992)
Magnesium (mg/day)	1675	294 (244, 350)
Zinc (mg/day)	1675	9 (7, 11)

*BMI* body mass index, *HRT* hormone-replacement therapy

^a^Physical activity level is defined as an individual’s total energy expenditure over 24 h, divided by their basal metabolite rate and is unitless

^b^National Deprivation Category based on postcode classification where “1” represents most affluence/least deprived and “6” represents least affluent/most deprived

BMD was measured using dual-energy X-ray absorptiometry (DXA; Lunar iDXA, GE Healthcare, Madison, WI, USA) at the femoral neck (FN) and L1–L4 lumbar spine (LS). Encapsulated spine phantoms were measured daily. A plot of phantom measurements showed an upward shift of 0.7%; BMDs measured after the phantom shift (*n* = 193) were adjusted for this shift.

All procedures involving human participants were approved by the East of Scotland Research Ethics Service. Written informed consent was obtained from all participants.

### Measurement of confounding factors

Physical activity level (PAL) was obtained using the same questionnaire as in the Scottish Heart Healthy Study [19]. PAL is calculated from the duration and intensity of activity performed in a 24-h period divided by basal metabolic rate; these were assessed for working and non-working days [20]. National deprivation category was assigned from postal codes in 1997–2000; the lowest number denotes the most affluent/least deprived and is used as a measure for socioeconomic status [21]. Questionnaires were used to assess social and demographic information including age, menopause status (pre-, peri-, post-menopausal), hormone-replacement therapy (HRT; past user, present user) use and smoking status (yes/no).

### Statistical analyses

All analyses were conducted using Statistical Analysis Systems statistical analysis package ver. 9.3 (SAS Institute, Inc.; Cary, NC, USA). Participant characteristics were described by means and standard deviations (SD) or medians and interquartile ranges (IQR) for continuous variables and percentages for categorical variables. Non-normally distributed variables were natural log-transformed prior to analysis.

The PLS method was used to extract successive, orthogonal, linear combinations (“dietary patterns” or “factors”) of the predictor (i.e. the 37 food groups) and response variables (i.e. nutrients associated with bone) to maximize the covariance between them (described in greater detail by Hoffman et al. [13]). The resulting factors are uncorrelated with one another and can be concurrently used in regression models without risk of confounding each other.

Response variables were chosen for their relationship with bone health and include dietary intakes of calcium, vitamin D, vitamin C, protein, alcohol, potassium, magnesium, phosphorus and zinc.

Food groups and nutrient response variables were adjusted for energy intake using the residual method [5]. We fit the 37 food groups as predictor variables and included the nine described dietary nutrients as response variables to obtain the dietary patterns (factors). Split-sample cross-validation with van der Voet’s test and inspection of correlation plots between the predictor and response scores were used to determine the number of factors to retain [22].

The retained factors were entered as continuous variables in regression models with continuous BMD as the outcome. Unadjusted and multivariable regression models were constructed separately for LS and FN BMD, adjusting for age, BMI, physical activity level, smoking status (current smokers/not) and national deprivation category (category 6 [least affluent/most deprived] as reference). Factors that were significant in these models were additionally categorized into quartiles to explore potential non-linear relationships. All *P* values are two-sided. We tested LS and FN BMD for trend across quartiles of the dietary patterns.

Additional adjustment for previous HRT use (*n* = 1259) and the interaction between the selected dietary patterns and previous HRT use were initially included but were not significant and not retained in the final model. Sensitivity analyses excluding participants with energy intake <3.35 or >14.65 MJ (*n* = 20), and participants with rheumatoid arthritis (*n* = 40), osteoarthritis (*n* = 358), other unspecified bone diseases (*n* = 18), previous bisphosphonates use *n* = 14) or oral steroid use (*n* = 44) did not markedly alter effect estimates. Regression models without energy-adjustment for food groups were explored but did not result in differences in results from regression modelling and were left adjusted.

## Results

Participant characteristics are shown in Table 1. Our study participants, on average, were 66 years old (SD 2.2) and overweight, consumed 10.1 MJ/day and had a LS BMD of 1.09 (0.17) and 0.93 g/cm^2^ (0.12) for FN BMD.

**Fig. 1:**
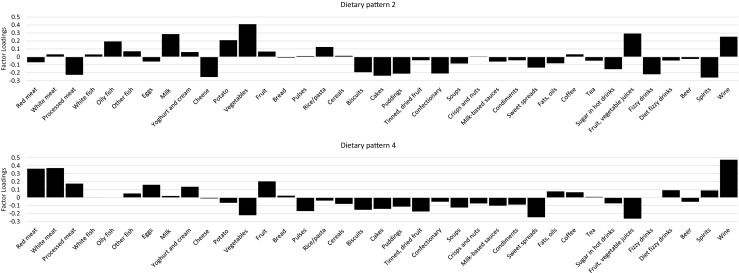
Factor loadings of food groups from dietary patterns significantly associated with lumbar spine or femoral neck bone mineral density derived using partial least-squares

Five factors were retained from the PLS analysis. These five factors explained 25% of the variation in the food groups and 77% of the variation in the response variables (Table 2). The main food group loadings for factors 2 and 4 are shown in Fig. 1. Factor loadings, which are the correlations between the derived factors and the food groups, were considered important when values were ≥0.2 using absolute values [13, 23]. A higher factor loading for a food item indicates greater contribution in constructing the factor and is used to interpret the composition of the factor. Factor loadings for factor 2 were shown to be primarily characterized by high intakes of fluid dairy, potatoes, vegetables, fruit and vegetable juices, and wine, and low consumption of processed meats, cheese, cakes, puddings, confectionary, fizzy/carbonated drinks, and spirits. Factor 4 was characterized by high intakes of red and white meats, fruits, and wine, and low intakes of vegetables, sweet spreads, and fruit and vegetable juices.

**Table 2:** Explained variation in food groups and responses from the first five dietary patterns derived from partial least-squares

	Explained variance in food groups^a^		Explained variance in responses^b^	
	Current	Total	Current	Total
Factor 1	9.2	9.2	49.4	49.4
Factor 2	5.6	14.8	10.1	59.5
Factor 3	3.8	18.6	6.7	66.2
Factor 4	3.3	21.9	5.9	72.1
Factor 5	3.2	25.1	5.2	77.3

^a^Foods from the food frequency questionnaire were aggregated into 37 good groups: red meat, white meat, processed meat, white fish, oily fish, other fish, eggs, milk, yogurt and cream, cheese, potato, vegetables, fruit, bread, pulses, rice/pasta, cereals, biscuits, cakes, puddings, tinned, dried fruit, confectionary, soups, crisps and nuts, milk-based sauces, condiments, sweet spreads, fats and oils, coffee, tea, sugar in hot drinks, fruit and vegetables juices, fizzy drinks, diet fizzy drinks, beer, spirits and wine

^b^Responses are dietary intakes of alcohol, protein, vitamin D, vitamin C, calcium, magnesium, zinc, phosphorus and potassium

**Fig. 2:**
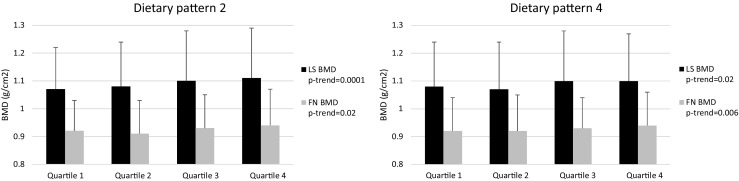
Mean and standard deviation of lumbar spine and femoral neck bone mineral density for quartiles of dietary pattern factors 2 and 4

In multivariable models, two of the factors were significantly associated with LS or FN BMD (Table 3). In adjusted analyses, a 1-unit increase in factor 2 was associated with a 0.012 g/cm^2^ higher LS BMD (95% CI: 0.006, 0.01) and 0.006 g/cm^2^ (95% CI: 0.002, 0.01) higher FN BMD, while a unit increase in factor 4 was associated with a 0.008 g/cm^2^ (95% CI: 0.003, 0.01) increase in FN BMD. When examining the relationship between quartiles of factors 2 and 4 with BMD, LS and FN BMD increased significantly across quartiles of both factor 2 (LS BMD: *P-*trend = 0.0001; FN BMD: *P-*trend = 0.02) and factor 4 (LS BMD: *P-*trend = 0.02; FN BMD: *P-*trend = 0.006) (Fig. 2).

**Table 3:** Association between a unit change in dietary patterns with lumbar spine and femoral neck bone mineral density

	LS BMD (g/cm^2^)						FN BMD (g/cm^2^)					
	Unadjusted			Adjusted^a^			Unadjusted			Adjusted^a^		
	*β*	95% CI	*P* value	*β*	95% CI	*P* value	*β*	95% CI	*P* value	*β*	95% CI	*P* value
Factor 1	−0.003	−0.007, 0.001	0.11	−0.0003	−0.004, 0.003	0.88	−0.005	−0.007, −0.002	<0.01	−0.001	−0.004, 0.001	0.37
Factor 2	0.009	0.003, 0.01	0.002	0.012	0.006, 0.01	<0.0001	0.004	−0.001, 0.01	0.09	0.006	0.002, 0.01	<0.01
Factor 3	−0.004	−0.01, 0.002	0.21	−0.004	−0.01, 0.002	0.26	0.003	−0.003, 0.007	0.31	0.003	−0.001, 0.007	0.18
Factor 4	0.008	0.0009, 0.01	0.03	0.007	0.00001, 0.01	0.05	0.01	0.005, 0.01	<0.01	0.008	0.003, 0.01	<0.01
Factor 5	0.005	−0.002, 0.01	0.18	0.004	−0.003, 0.01	0.21	0.005	−0.0004, 0.01	0.07	0.003	−0.002, 0.007	0.22

*LS* lumbar spine, *BMD* bone mineral density, *FN* femoral neck, *CI* confidence interval

^a^Adjusted for age, BMI, physical activity level, smoking status, national deprivation category

## Discussion

In this study assessing the use of a novel data-reduction technique to derive dietary patterns in relation to bone health in post-menopausal women, we observed two dietary patterns that were positively associated with LS and FN BMD. These two dietary patterns, or factors, are described by foods that are considered components of a “healthy” diet: intakes of fruits, vegetables, milk and wine, and low intakes of processed meats, cheese, cakes and sweets, fizzy/carbonated beverages and spirits.

Similar to other studies, we found that consumption of alcohol, in the form of wine, was positively associated with BMD, which has been observed in other studies [24]. Moderate alcohol consumption may be beneficial by raising serum estradiol levels [25] and stimulating secretion of calcitonin [26], which has been associated with decreased rate of vertebral fractures and increased bone mass. Wine, rather than spirits, may be valuable to bone health because it is a source of boron, which supports bone growth and maintenance, possibly through enhanced angiogenesis or signal transduction [27, 28].

Interestingly, vegetables and fruit and vegetable juices were negatively correlated with factor 4, one of the two dietary patterns positively associated with BMD. This perhaps suggests that other elements of this dietary factor, such as fruit intake, may have offset the negative loading of vegetables and fruit and vegetable juices to result in the overall beneficial relationship with BMD. A similar explanation was proposed for the loading of a partially hydrogenated soybean oil onto a “vegetable” dietary pattern associated with a decrease in risk of myocardial infarction [29]. The authors hypothesized that the potentially atherogenic effects of partially hydrogenated fatty acids may have been counteracted by the beneficial elements in the vegetable dietary pattern.

Dairy intake also differed in how it loaded on factor 2, which was characterized by high intakes of fluid dairy but low intakes of cheese. Włodarek et al. [30] observed a similar situation, with positive correlation between milk intake and BMD, but negative correlation with rennet and cottage cheeses. Calcium may be the major nutrient attributed to dairy products in relation to bone health but its absorption is promoted, and therefore influenced, by lactose [31]. The authors hypothesized that, as lactose content is higher in milk than in cheeses, the increased calcium absorption led to the positive association with BMD. But, as calcium intake in our population was above the reference nutrient intake (RNI) in the UK of 700 mg/day for adults [20], we postulate that our finding may also be a result of how our food groups were constructed.

We separated dairy products into three groups: milk drinks (including dried, condensed, soy), yogurt and cream products (including full-fat, low-fat, skimmed), and cheese products (including full-fat, low-fat, hard, soft) in order to differentiate between the dairy products. There is no consensus for how to group foods when conducting a dietary pattern analysis; studies subjectively cluster foods based on similarities in food and nutrient intake, culinary preference, and logic [32]. How other studies chose to categorize dairy products may be why positive associations between dairy and bone health were observed when all dairy products were grouped together [33], while inconsistent relationships were found when dairy products were in multiple sub-groups [9].

This is the first study to apply PLS in determining dietary patterns and their relation to bone; there are two other studies applying a statistical method that takes nutrient or biomarker response variables into consideration when identifying dietary patterns, through the use of RRR [11, 34]. In a study of Australian adolescents by van den Hooven et al. [11], patterns were characterized based on whether the response variables (protein, calcium and potassium) were high or low. Two patterns were extracted: pattern 1 with high protein, calcium and potassium, and pattern 2 with high protein, low calcium and potassium. Only the first pattern was positively associated with BMD and bone mineral content; the foods that loaded on this pattern were comparable to a healthy dietary pattern characterized by high intakes of low-fat dairy, whole grains, vegetables, fish, fruit, legumes and low intakes of confectionary, chips/crisps, sweets and processed meats. The study by Ward et al. [34] utilized food diaries collected from age 36 years to 60–64 years to determine RRR dietary patterns constructed with protein, calcium, and potassium as response variables, and how dietary patterns may track through time. The first dietary pattern extracted when participants were 36 years old was positively associated with dietary intakes of fruits and vegetables and low-fat dairy (milk and yogurt), while negatively associated with intakes of refined grain products, processed and sugary foods, and alcohol. Adherence to this dietary pattern at each subsequent visit was calculated for each individual, and the trajectories showed that an adherence to the dietary pattern was positively associated with bone health at age 60–64 years. These two studies utilizing another dietary data reduction technique also showed that a healthy, nutrient-dense diet is beneficial for bone health in both young and older participants.

Other studies deriving non-PLS dietary patterns in older adults also found healthy or nutrient-dense dietary patterns to be associated with decreased bone resorption [10], decreased fracture risk [8, 35] or increased BMD [12], and dietary patterns with high loadings for energy-dense, processed foods were associated with lower BMD [9, 10, 12]. While some studies report null or contradictory relationships between healthy dietary patterns and bone health [36], adherence to dietary patterns which are nutrient-dense are beneficial for health outcomes beyond bone, including hypertension [37] and type 2 diabetes [38].

Strengths of our study include a large sample size and the use of bone-related nutrient biomarkers as response variables in the novel PLS procedure to construct dietary patterns. The benefit of using PLS, as opposed to other data reduction techniques, results from including knowledge about the intermediary response variables between the food groups and the health or disease outcome. Studies differ in what intermediary response variables to include. We included calcium and vitamin D, as they are necessary for calcium absorption and bone formation; in postmenopausal women, calcium intake was positively associated with FN BMD change and hypothesized to reduce bone loss [24] and there is good evidence to suggest that intakes of combined calcium and vitamin D are beneficial for BMD [39]. Vitamin C is an antioxidant that could reduce bone loss by counteracting oxidative stress that may reduce BMD, and dietary intakes have been associated with increased BMD [40]. Protein is necessary for many bone-related activities including growth factors and hormones that impact bone synthesis, break-down, and bone matrix structure [41]. Moderate alcohol intake has been positively associated with BMD and less bone loss by promoting secretion of calcitonin or increasing endogenous oestrogens [24]. Phosphorus is necessary for mineralization of the skeleton and inadequate levels result in impaired bone integrity and can lead to osteomalacia [42]. Magnesium may influence bone metabolism through its necessity as a co-factor in metabolism and enzymatic processes and, directly, it may decrease hydroxyapatite crystal size [43]. Zinc is necessary for collagen synthesis and osteoblastic activity, and a trial among postmenopausal women showed that supplementation resulted in a small increase in BMD over a 2-year period [43, 44]. Finally, potassium is hypothesized to benefit bone by producing an alkaline environment, reducing the need to recruit skeletal calcium salts to counteract the acids generated from acid-generating foods [3, 43].

The PLS approach in including response variables results in dietary factors that are underpinned by the underlying nutrients of interest, so the choice of the intermediary response variables will influence how dietary factors are constructed. While RRR is similar to PLS, dietary patterns identified by RRR are limited to those nutrients included as the intermediary variables and may miss out on dietary patterns that specify nutrient pathways not included as intermediary response variables. Therefore, the dietary patterns produced by RRR are more tailored to the health outcome in question, whereas the PLS procedure is a better tool for informing how to modify intake of dietary intake to elicit a nutrient response and impact health outcomes. While Hoffman et al. [29] concluded that RRR was better at identifying dietary patterns in relation to diabetes risk, DiBello et al. [29] found that PCA and PLS produced more dietary patterns associated with their outcome of myocardial infarction risk. Thus, our objective was methodologically driven by the use of this novel technique to assess how dietary patterns may be derived. The dietary patterns we identified support previous findings using other dietary reduction techniques, and this present analysis using a different data reduction technique confirms and adds to the evidence of the benefits of a healthy dietary pattern.

This study also had several limitations. Like other data-reduction methods, the assessment of dietary intake is subject to measurement error and residual confounding, even though our FFQ had been previously validated using weighed food records and biochemical markers of antioxidant status [5]. It is also possible that the dietary patterns observed were based on nutrients that were chosen as our response variable nutrients, where different nutrient response variables may elicit formation of different dietary patterns. Additionally, dietary patterns extracted in one population with data-reduction methods will not reproduce the same dietary factors with the same food loadings in another data set, and thus, our results are limited to postmenopausal women from northeast Scotland. Another limitation is the cross-sectional nature of our study, where causality cannot be assumed.

Nevertheless, multiple studies have reported general “healthy” dietary patterns that have similar food group loadings. The persistent relationships between similar dietary patterns with various outcome measures have led government advisory groups such as the US Dietary Guidelines Advisory Committee to recommend that the population achieve optimal health through a healthy diet, rather than focusing on specific foods or nutrients [7]. Earlier initiatives had also attempted to move from nutrient population goals to one that is foods-based [45].

In summary, dietary pattern analysis using the PLS method extracted dietary patterns rooted in a biological link between dietary intakes and the health outcome; this is important because foods are the modifiable aspect in this relationship. In this study, we observed that adherence to dietary patterns with higher intakes of nutrient-rich foods and lower intakes of energy-dense foods was associated with an increase in bone mineral density in a population of postmenopausal women. Our results show that PLS is an appropriate method to determine which dietary patterns are associated with BMD and supports previous findings using other data-reduction techniques on the relationships between diet and health.

### Authorship

T.C.Y., H.M.M. and G.G.D. contributed to the conception and design of the research, T.C.Y. analysed and wrote the manuscript, H.M.M. and G.G.D. obtained funding for the research and supervised research direction, L.S.A. and H.M.M. supervised analysis and interpretation. All authors reviewed the manuscript and approved the final version.

## Electronic supplementary material

ESM 1 (PDF 151 kb)
