# Supplementary figures and images for: An application of partial least squares for identifying dietary patterns in bone health
Source: Arch Osteoporos. 2017 Jul 12;12(1):63. doi: 10.1007/s11657-017-0355-y (PMC5506508; doi:10.1007/s11657-017-0355-y)

Supplementary Figure 1. Factor loadings for dietary patterns 1, 3, and 5.

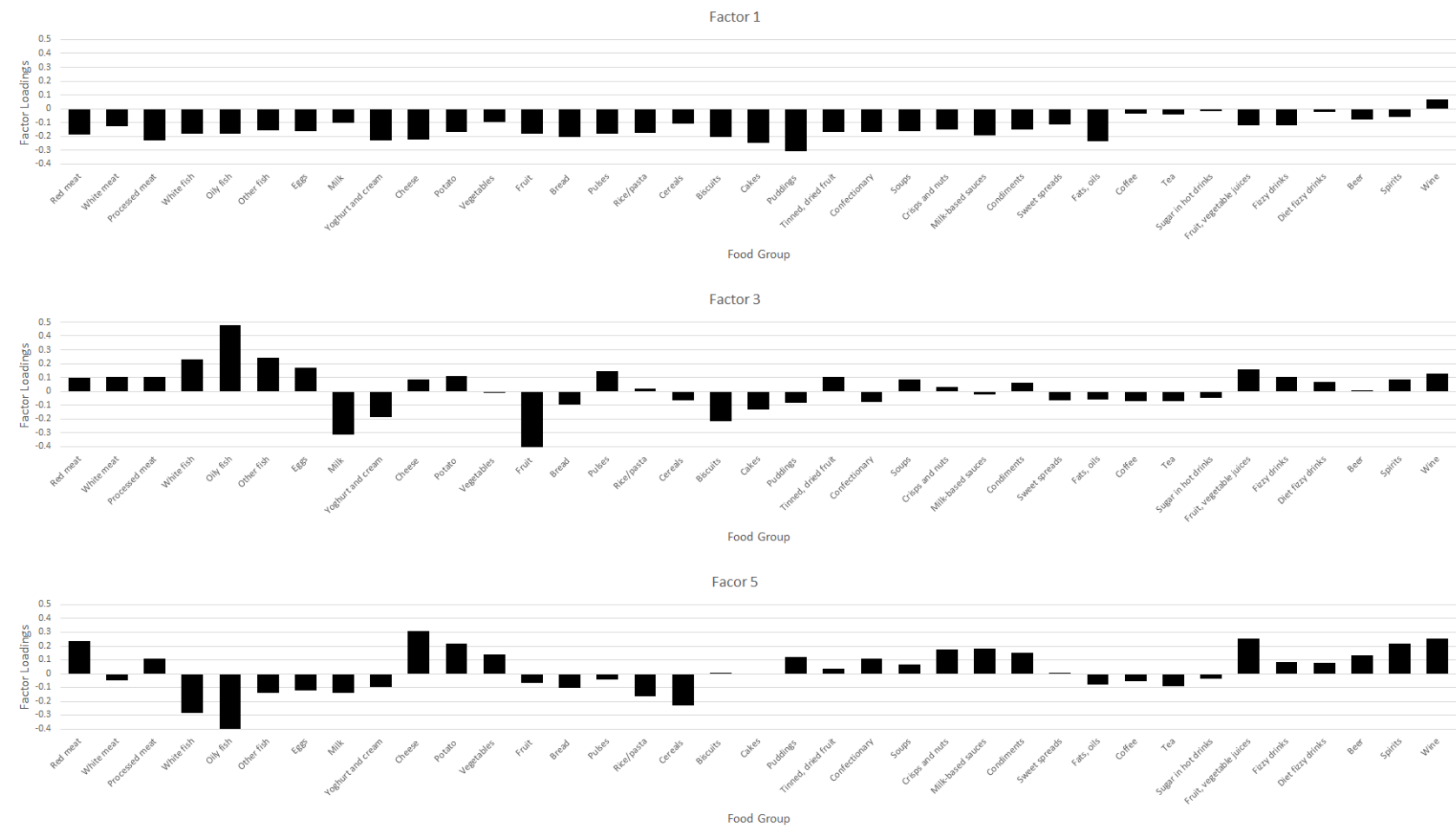

Supplement: Supplementary file 1 — (PDF 151 kb) [file 11657_2017_355_MOESM1_ESM.pdf]
